# Real-time investigation of protein unfolding at an air–water interface at the 1 s time scale

**DOI:** 10.1107/S0909049513023741

**Published:** 2013-10-02

**Authors:** Yohko F. Yano, Etsuo Arakawa, Wolfgang Voegeli, Tadashi Matsushita

**Affiliations:** a Department of Physics, Kinki University, 3-4-1 Kowakae, Higashiosaka City, Osaka 577-8502, Japan; b Department of Physics, Tokyo Gakugei University, Koganei, Tokyo, Japan; c Photon Factory, Institute of Materials Structure Science, KEK, Tsukuba, Ibaraki, Japan

**Keywords:** protein unfolding, air–water interface, X-ray reflectivity, time-resolved measurements

## Abstract

Protein unfolding at an air–water interface has been demonstrated such that the X-ray reflectivity can be measured with an acquisition time of 1 s using a recently developed simultaneous multiple-angle–wavelength-dispersive X-ray reflectometer. This has enabled the electron density profile of the adsorbed protein molecules to be obtained in real time. A globular protein, lysozyme, adsorbed at the air–water interface is found to unfold into a flat shape within 1 s.

## Abstract 2

Protein unfolding at an air–water interface is followed in real time by a recently developed simultaneous multiple-angle–wavelength-dispersive X-ray reflectometer with a time resolution of 1 s.

## 1. Introduction

Structures and structural evolutions of proteins during folding–unfolding carry critical information for understanding correlations between structures and functions of the proteins. Many approaches to follow the structural changes occurring in the folding processes have been used previously, including time-resolved small-angle X-ray scattering combined with continuous-flow techniques (Kathuria *et al.*, 2011 ▶). In those studies, protein folding initiated by dilution of denaturant concentrations, changing pH or a temperature jump, is followed on the sub-millisecond time scale.

Another approach towards understanding the mechanisms of protein folding is to study the kinetics that lead to protein unfolding under various conditions. The conformation of protein molecules is determined by a balance of various forces, including van der Waals attraction, electrostatic interaction, hydrogen bonding and conformational entropy. When protein molecules encounter interfaces, they are often adsorbed on the interface. The conformation of an adsorbed protein molecule strongly depends on the interaction between the protein and the interface. The major driving forces of protein adsorption at an air–water interface are hydrophobic interactions and they induce protein unfolding (Yano, 2012 ▶). Previously, time-resolved measurements with a time resolution of 3 min have been performed by an angle-scan X-ray reflectometer to investigate the adsorption process of a globular protein lysozyme, on an air–water interface. It was found that the lysozyme molecule initially adsorbed by adopting a flat unfolded structure. In contrast, as adsorption continues, the lysozyme molecules gradually adapt their configuration to the surroundings (Yano *et al.*, 2009 ▶, 2010 ▶). However, more rapid measurements are necessary for investigating the early unfolding events at an air–water interface.

In the present work, we investigate the protein adsorption at an air–water interface with a time resolution of 1 s using a recently developed simultaneous multiple-angle–wavelength-dispersive X-ray reflectometer (Matsushita *et al.*, 2013 ▶).

## 2. X-ray reflectivity

Using X-ray reflectivity, the electron density profile along the interface normal can be obtained since the wavevector transfer **q** = **k**_out_ − **k**_in_, which is determined by the incident and outgoing X-ray beams, is perpendicular to the interface. In the angle-scanning method, the reflected intensity of monochromatic X-rays is recorded as a function of the incident angle. Even when synchrotron radiation has been used, breaking the minute barrier in time resolution has been difficult to achieve so far. For studying the structural kinematics or dynamics of samples, it would be better to be able to simultaneously measure the whole profile of the X-ray reflectivity curve with higher time resolution.

### 2.1. Simultaneous multiple-angle–wavelength-dispersive X-ray reflectometer

**Figure 1:**
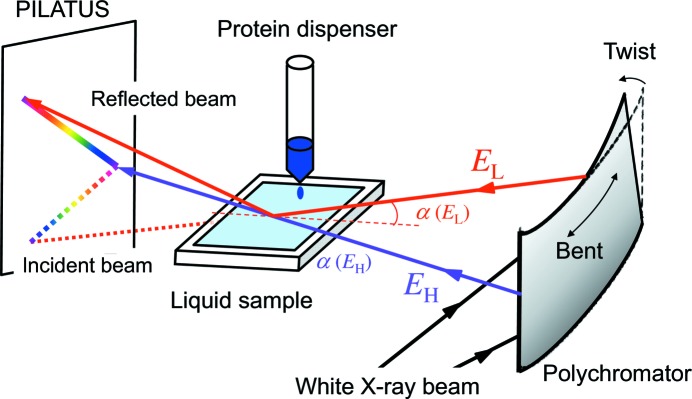
Schematic figure of the DXR combined with the protein injection system.

The X-ray reflectivity measurements were performed using the recently developed simultaneous multiple-angle–wavelength-dispersive X-ray reflectometer (DXR) (Arakawa *et al.*, 2013 ▶; Matsushita *et al.*, 2013 ▶) shown in Fig. 1 ▶. A synchrotron white X-ray beam from a bending magnet is incident on the polychromator crystal. The polychromatic X-ray beam is focused onto the sample with incident angles α that change continuously between α(*E*_H_) and α(*E*_L_). The intensity distribution of the specularly reflected beam was measured by using a PILATUS 100K detector.

### 2.2. Theoretical background

The X-ray reflectivity for liquid surfaces is described by the Born approximation (Perhsan & Schlossmann, 2012 ▶),
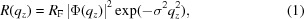
where *R*_F_ is the Fresnel reflectivity for an ideally flat interface, σ is the roughness of the interface and 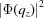 is the intrinsic structure factor normal to the surface, which is expressed as
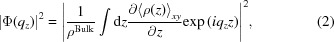
where 〈ρ(*z*)〉_*xy*_ is the lateral average electron density profile. These formulas can also be derived using the Parratt formalism which considers a fully dynamical theory of wave propagation in a stack of layers separating a semi-infinite layer of bulk material from a semi-infinite vapour layer (Parratt, 1954 ▶). In the present study, the X-ray reflectivity data were fitted using a two-slab model with the *Parrat32* software (Braun, 1997 ▶) based on the Parratt formalism with the thickness, electron density and roughness of the two slabs as parameters.

## 3. Experimental methods

### 3.1. X-ray reflectivity measurements

The X-ray reflectivity measurements were performed using the simultaneous DXR described above in §2.1. A white synchrotron beam from a bending-magnet source of the 6.5 GeV electron storage ring PF-AR at KEK was incident onto the bent-twisted polychromator crystal to obtain the wavelength region of 0.561–0.648 Å (energy 22.1–19.1 keV) and incident angles of 0–1.7°.

### 3.2. Protein injection

The protein injection system is schematically shown in Fig. 1 ▶. A protein solution of 1 cm^3^ was injected in 0.3 s using a liquid dispenser (Ultra Dispensing Stations 30 PSI, San-Ei Tech) into a 42 cm^3^ phosphate buffer solution of pH 7 contained in a Langmuir trough to give a final protein concentration of 1 mg ml^−1^ (Yano *et al.*, 2009 ▶). The Langmuir trough was covered with an acrylic hood to suppress liquid evaporation. The X-ray reflectivity measurements were started before injection of the protein solution. The data collection time was 1.0 s. The position of the X-rays on the sample was changed horizontally at intervals of 1 s to avoid radiation damage.

### 3.3. Protein sample

A globular protein, lysozyme (hereafter, LSZ), was used as the protein sample. LSZ has an elliptical shape with approximate dimensions of 30 Å × 30 Å × 45 Å. The isoelectric point is 11.35. Three-times crystallized and lyophilized hen egg-white lysozyme was purchased from Sigma (product No. L6876) and used as supplied. Protein solutions were made using a phosphate buffer solution (0.02 *M* NaH_2_PO_4_/Na_2_HPO_4_) of pH 7 (ionic strength: 0.02 *M*) using UHQ-grade water.

## 4. Results and discussions

### 4.1. Detector images

**Figure 2:**
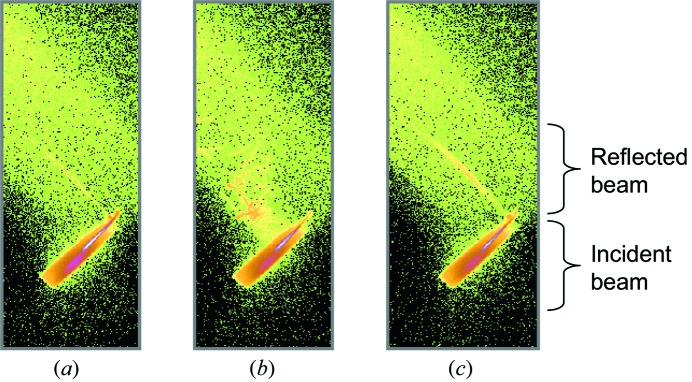
Detector images of the X-ray beam reflected by the phosphate buffer solution: (*a*) before protein injection, (*b*) 2 s and (*c*) 12 s after protein injection.

Fig. 2 ▶ demonstrates typical detector images of the phosphate buffer solution measured before and after the protein injection. Within 10 s after protein injection, the X-ray beam was not ideally reflected because of the motion of the liquid surface. Otherwise, the reflected beam forms straight lines as expected.

### 4.2. X-ray reflectivity curves

**Figure 3:**
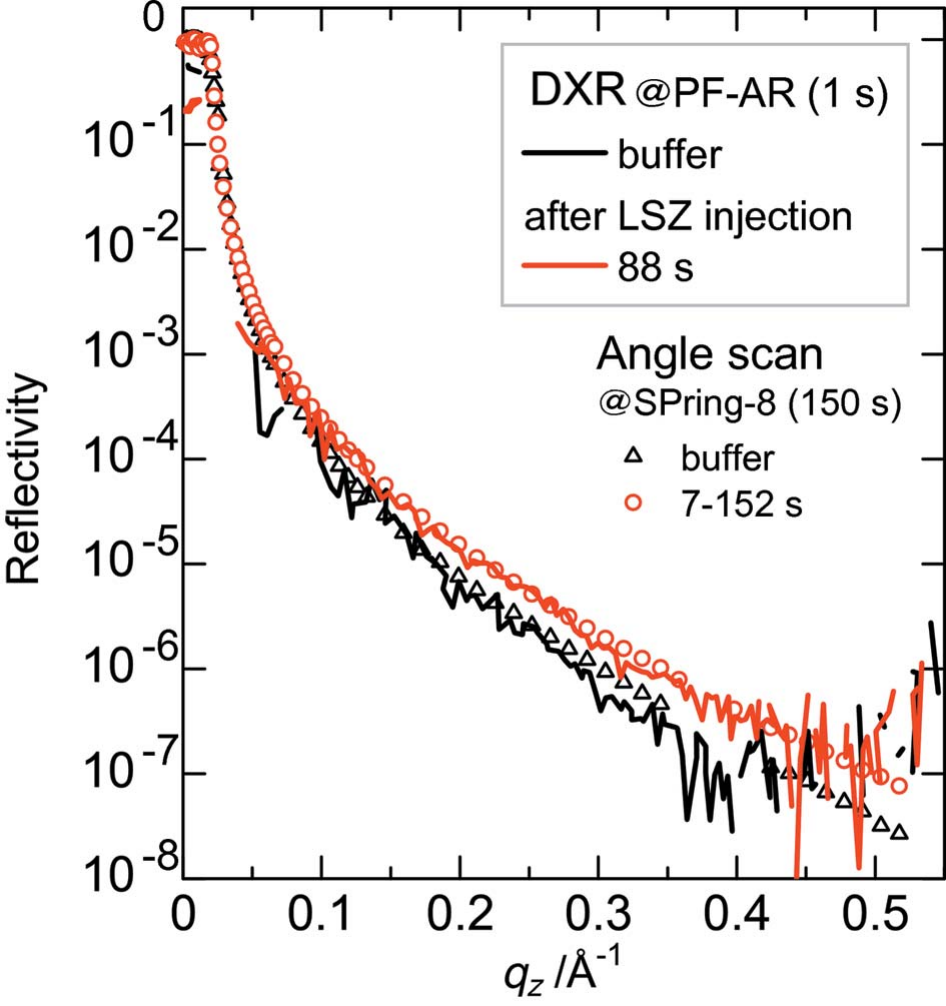
X-ray reflectivity curves of the phosphate buffer solution before and after protein injection. DXR: before (black lines) and 88 s after (red lines) LSZ injection. Angle scan: before (triangles) and 7–152 s after (circles) LSZ injection.

Fig. 3 ▶ shows X-ray reflectivity curves of the phosphate buffer solution before and after protein injection. The reflectivity curves previously measured by the angle-scan reflectometer at SPring-8 (Yano *et al.*, 2009 ▶) are also shown for comparison. In the case of the angle-scan reflectometer, the time required for each scan was 150 s with an integration time at each angle of 1 s. X-ray reflectivity measurements were started 7 s after injection of the protein and finished at 152 s. With the present reflectometer, the whole profile of the X-ray reflectivity curve down to a reflectivity of 10^−7^ can be obtained in 1 s, showing good agreement with those obtained by the angle-scan reflectometer. Higher signal/noise ratio and time resolutions would be attained by using a more intense X-ray source.

### 4.3. Time-resolved measurement of a protein adsorbed at an air–water interface

**Figure 4:**
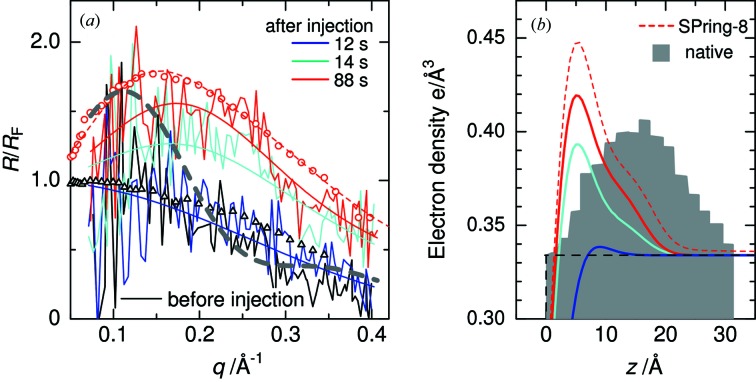
(*a*) Time dependence of X-ray reflectivity profiles. The data were divided by the Fresnel reflectivity of the air/buffer interface. The continuous lines are the fit to the data. The triangles and circles are the data measured by the angle-scan reflectometer at SPring-8 before and 7–152 s after LSZ injection, respectively. The dashed gray curve represents the simulated profile of native LSZ whose electron density profile is shown in (*b*). (*b*) Electron density profiles corresponding to the fits to the data. The dashed lines are those for 7–152 s after protein injection measured by the angle-scan reflectometer. The gray area represents the simulated profile of native LSZ with a side-on orientation (Tiemeyer *et al.*, 2010 ▶).

Fig. 4 ▶ shows the time dependence of the X-ray reflectivity profiles divided by the Fresnel reflectivity *R*_F_, along with electron density profiles obtained by fitting the data. The profile at 88 s shows good agreement with that measured by the angle-scan reflectometer, indicating that the protein injection was reproducible. The X-ray reflectivity profile remains unchanged with that of the buffer solution until 12 s after LSZ injection and then increases rapidly in 2 s. We think the initial adsorption of LSZ has started at 12 s when the motion of the liquid surface was stopped. The dashed gray curve represents the simulated reflectivity of native LSZ with a side-on orientation. Obviously, the observed reflectivity curves are inconsistent with the simulated curve. As shown in Fig. 4(*b*) ▶, the initially adsorbed LSZ at 14 s has a dense upper slab 6 Å thick and a lower slab 10 Å thick, indicating that the protein is highly denatured in comparison with the globular structure that is observed in the bulk (gray area). This configuration does not change with increasing adsorption amount of LSZ. We conclude, therefore, that the unfolding event of LSZ under the present conditions completes within 1 s. It is known that at lower protein concentrations the lower adsorption rate induces larger conformational changes of adsorbed protein molecules. In the case of LSZ, the initial adsorption rate from a 0.01 mg ml^−1^ LSZ solution is about 100 times slower than that from the 1 mg ml^−1^ solution (Yano *et al.*, 2009 ▶). It is worth investigating whether the unfolding transition is slower under lower protein concentrations.

## 5. Conclusions

The protein adsorption process at an air–water interface was observed in real time with a time resolution of 1 s using a recently developed simultaneous multiple-angle–wavelength-dispersive X-ray reflectometer. Although it was not possible to obtain the X-ray reflection curves within 10 s after the protein injection, we have succeeded in observing the initial event of the protein adsorption. The obtained electron density profile for the initially adsorbed lysozyme molecules is highly distorted as compared with the native configuration indicating a flatter structure at the interface and this configuration does not change with increasing adsorption amount. The present results suggest that the lysozyme molecules initially adsorbed at an air–water interface unfold within 1 s. Higher time resolution would be attained by reducing the dead-time associated with the protein injection and using a more intense X-ray source.
